# Patient and hospital staff perspectives on introducing pharmacist-led medication reviews at an orthopedic ward: a mixed methods pilot study

**DOI:** 10.1007/s11096-025-01874-7

**Published:** 2025-02-20

**Authors:** Joo Hanne Poulsen Revell, Maja Schlünsen, Abisha Kandasamy, Annette Meijers, Jens Eggers, Lene Juel Kjeldsen

**Affiliations:** 1 The Hospital Pharmacy, University Hospital of Southern Denmark, Kresten Philipsens Vej 15, 6200 Aabenraa, Denmark; 2 The Hospital Pharmacy Research Unit, University Hospital of Southern Denmark, Kresten Philipsens Vej 15, 6200 Aabenraa, Denmark; 3 The Department of Regional Health Research, University of Southern Denmark, Odense, Denmark; 4 The Faculty of Health Sciences, University of Southern Denmark, Odense, Denmark; 5 Department of Orthopaedics, University Hospital of Southern Denmark, Aabenraa, Denmark

**Keywords:** Polypharmacy, Patient safety, Medication review, Clinical pharmacy, Orthopedic Ward

## Abstract

**Background:**

Multi-morbidity is associated with multiple medication use, which potentially increases the risk of adverse drug events. Pharmacist-led medication reviews have been introduced to meet these challenges.

**Aim:**

To evaluate the implementation of pharmacist-led medication reviews for older patients admitted to an orthopedic ward in terms of quality and safety from the perspectives of patients, hospital-based physicians, nurses, and healthcare assistants.

**Method:**

Patients (n=11) were interviewed, with the interviews having a reflexive thematic analysis using the hermeneutic approach, while healthcare-professionals’ (HCPs) (n=26) perspectives on pharmacist-led medication reviews were assessed using questionnaires.

**Results:**

The qualitative patient interviews revealed four themes: (1) Positive perception of pharmacists’ medication communication, (2) Mixed perceptions of a medication review, (3) Satisfaction with the general outcome of the medication review, and (4) Safety perception with medication treatment. Twenty-six HCPs completed the questionnaire (response rate 48%) with a distribution of 10 hospital-based physicians (38%), eight nurses (31%), and eight healthcare assistants (31%). Almost 85% of the HCPs (n = 22) were familiar with the pharmacist conducting medication reviews. More than 70% of the HCPs reported that pharmacist-led medication reviews contributed to increased quality of admitted patients’ medication use.

**Conclusion:**

High levels of satisfaction with the outcomes of the medication reviews—particularly regarding quality, patient safety, and their overall positive impact on the ward—indicate that both patients and HCPs perceived the service as highly valuable in supporting patient care throughout the medication process.

**Supplementary Information:**

The online version contains supplementary material available at 10.1007/s11096-025-01874-7.

## Impact statements

- Patients may experience difficulty recalling the exact content of a medication review conversation, which indicates that the timing and content of such a conversation should be considered carefully to ensure benefit for the patient.
- Healthcare professionals assessed the conducted medication reviews to increase the quality of medication treatment and to increase patient safety. Hence, the service was considered a valuable addition to patient care.
- Hospital-based physicians preferred written medication notes instead of verbal communication, emphasizing the physicians' limited availability during the day due to operations, thereby limiting availability for verebal communication.

## Introduction

Lack of medication treatment coordination, especially among multi-morbid patients, results in suboptimal medication treatment, medication errors, hospital admissions and premature death [1, 2]. In addition, the aging population is a challenge to healthcare systems worldwide as older adults are vulnerable to non-communicable diseases and particularly multi-morbidity [1–3]. Multi-morbidity is associated with multiple medication use (polypharmacy), which potentially increases the risk of adverse drug events (ADEs) [4, 5]. Consequently, pharmacist-led medication reviews have been introduced to meet these challenges [6, 7].

Several studies have explored the effect of implementing pharmacist-led services in orthopedic wards, reporting positive outcomes such as preventing ADEs, improving medication safety, and supporting healthcare professionals (HCPs) in the medication process [8–11].

The orthopedic surgery setting is complex, involving many professionals and patient pathways through different units (anesthesia, intensive care, operating room) [9]. Thus, the risk of medication errors is increased due to pathway transitions, but when clinical pharmacists or pharmacy teams have been introduced to orthopedic wards, there is an overall increase in medication safety [9]. Studies show that introducing clinical pharmacists or pharmacy teams positively impacts patient care [6, 9, 12]. In addition, pharmacist-led medication reviews have been reported by hospital-based physicians to positively impact their daily workload, increase the quality of the medication discharge plans and contribute to a more rational prescribing practice at discharge [6, 9, 12]. Further, clinical pharmacists at hospital wards provide better data transmission between the hospital and the general practitioners (GPs) [9]. In addition, it has been suggested that pharmacists focus some recommendations specifically to the specialty of hospital-based physicians, whereas other recommendations should be directed towards the patient’s GP [10]. In this study, clinical pharmacists were introduced to an orthopedic setting in Denmark to perform medication reviews primarily to support hospital-based physicians at a small hospital in the medication process. This direct involvement of pharmacists in patient care is unusual at this ward, offering a unique opportunity to evaluate the implementation of pharmacist-led medication reviews in hospitals from the perspectives of hospital-based staff (physicians, nurses, and healthcare assistants).

Patient involvement in medication reviews contributes to the identification of drug-related problems, such as poor therapy control, non-optimal drugs and unintentional nonadherence [13]. Patients’ perceptions of pharmacist-led medication reviews have been explored in studies that report generally high patient satisfaction with the service [14–17]. However, potential issues have been reported, such as a lack of communication about the purpose of the medication reviews, the unclear role of pharmacists and patient unawareness of medication changes made during admission [14–18]. Further exploration of patients’ needs is necessary to improve pharmacist-led medication reviews and communication pathways.

### Aim

To evaluate the implementation of pharmacist-led medication reviews for older patients admitted to an orthopedic ward in terms of quality and safety from the perspectives of patients, hospital-based physicians, nurses, and healthcare assistants.

### Ethics approval

The data collected did not contain patient identification information and was not a biomedical study. Thus, approval from the National Ethics Committee was not required. Written consent was obtained from patients in the interviews regarding their satisfaction with the pharmacist-led interview review. The satisfaction questionnaire for HCPs was anonymous. Further, relevant approvals by the hospital and the orthopedic management in Hospital Sønderjylland allowed the study to proceed with approval to store data per regional policy (23/49779 and 23/52970).

The datasets generated during the current study have not been made publicly available due to personal identifiability but are available from the corresponding author upon reasonable request.

## Method

### Study design

This mixed method pilot study used reflexive thematic analysis with a hermeneutic approach to explore patients’ and HCPs’ perspectives on pharmacist-led medication reviews in an orthopedic ward. Qualitative face-to-face interviews were the most appropriate method to obtain in-depth knowledge about the patients' perspectives. Further, interviews create a confident and safe space between the interviewer and participant [19–21]. Questionnaires were chosen to evaluate the HCPs' attitudes towards pharmacist-led medication reviews, as the methodology is useful in describing the characteristics of a larger population, enhancing the generalizability of findings [19]. The reporting guideline “Good Reporting of A Mixed Methods Study (GRAMMS)” was applied [22].

### Setting

**Fig. 1:**
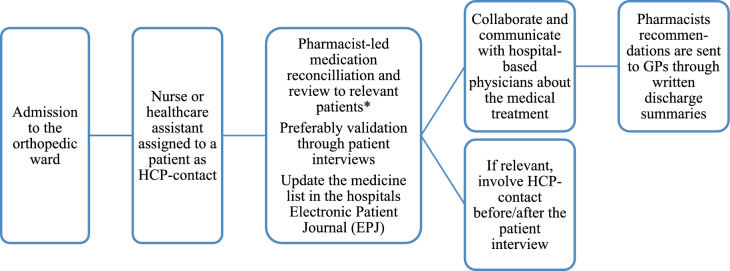
Patient admission and pharmacist-led intervention at the orthopedic ward. *Inclusion criteria (patients aged 65+ admitted with hip injury or hip fracture)

The study was conducted at an orthopedic ward comprising 30 beds at Hospital Sønderjylland. From April 2023 to March 2024, two clinical pharmacists conducted medication reviews for elderly patients (65 + years) admitted with hip injury or fracture. The role of the pharmacists was to support hospital-based physicians’ rational use of medication for admitted patients, and their interface with nurses and healthcare assistants was primarily to discuss relevant potential problems with the patient’s current medication. The clinical pharmacists were present 3–4 days weekly from 8 am to 2 pm. When patients self-administered their medication at home, the pharmacist provided information about any new medications during admission (Fig. 1).

The medication reviews were conducted before or after the surgery, depending on the patient’s convenience. The medication reviews included a conversation with the patient regarding their medication regimen. The medication reviews were performed using regional, national, and international recommendations e.g. treatment guidelines, national medication cessation list.

Two senior clinical pharmacists JHPR and AM performed the medication reviews, whereas MS (a junior pharmacist and PhD fellow) and AK (master thesis student in pharmacy) collected the qualitative and quantitative data, respectively.

### Study populations

#### Interviews

Face-to-face interviews were performed with eligible patients who had received a pharmacist-led medication review during admission between December 2023 and January 2024. The patients were purposively selected based on their ability to contribute and engage actively in an interview. Relevant patients were invited to participate in the interview. The interviews took place shortly after the medication review. The interviewer (MS) had no prior contact with the patients. All interviews were audio recorded and transcribed verbatim in Danish with written consent from the patients.

#### Questionnaire

An invitation to participate in an online evaluation questionnaire was sent to hospital-based physicians, nurses, and healthcare assistants at the orthopedic ward in May 2024. The participants were chosen based on their availability and willingness to participate in the study and their familiarity with pharmacist-led medication reviews.

### Data collection

#### Interview guide

A semi-structured interview guide was developed based on the aim of the study and existing literature (supplementary information S1) [14–17]. By applying a semi-structured guide, the interviewer could explore the research topic and follow up on topics if necessary. The interview guide was piloted amongst five patients; a few adjustments were necessary, and the guide was corrected accordingly.

#### Questionnaire

The evaluation questionnaire was designed to assess the perceived value of pharmacist-led medication reviews from HCPs’ perspectives using a 5-point Likert Scale (supplementary information S2). As the collaboration and communication between pharmacists and hospital-based physicians is more extensive than the pharmacists’ interface with nurses and healthcare assistants, the questionnaire aimed at the hospital-based physicians contained more questions than those directed at nurses and healthcare assistants. The online-based Research Electronic Data Capture system, RedCap, (version 13.7.18) was used to develop the questionnaire. The questionnaire was based on previous questionnaires (unpublished) that were initially developed to evaluate pharmacist-led medication reviews in Danish hospitals.

### Data analysis

#### Interviews

A reflexive thematic analysis developed by Braun and Clarke was used to identify key themes [23]. Two authors (JHPR and MS) performed the coding individually and discussed any discrepancies until a consensus was reached. JHPR and MS discussed and combined the codes into sub-themes and themes to ensure the analysis results were confirmable.

#### Questionnaire

Data were processed in Excel, and descriptive statistics were used to measure the frequency of responses and where appropriate cross-tabulations were applied.

## Results

### Qualitative interviews

In total, 11 patients were interviewed: three men and eight women. During one interview (P5 Male), it was discovered that the patient had not had a medication review, so this interview was not included in the analysis. All participants were 65 or above years of age and hospitalized due to hip injury or fracture, but concurrent medical conditions were not necessarily documented in the patient’s electronic chart. Partners/relatives of four patients were present during the interviews, and the interviews lasted, on average 19 min (range 13–29 min). The thematic analysis revealed four themes: (1) Positive perception of pharmacists’ medication communication, (2) Mixed perceptions of a medication review, (3) Satisfaction with the general outcome of the medication review, and (4) Safety perception of medication treatment. The four themes are described below.

#### Theme 1: positive perception of pharmacists’ medication communication

All participants expressed appreciation towards the conversation with the clinical pharmacist. They perceived the pharmacists as kind, good listeners, and easy to understand.

*“I was slightly informed about the various things… She could easily tell how it was. She was very sweet…”* [P4 Female].

*… I’ve talks to them many times and I think as long as I know what I’m getting, it’s just fine”* [P11 Male].

*"And it was nice, I had a good feeling afterwards… it was nice to be listened to and if there was something, well, then you could discuss it”* [P8 Female].

Furthermore, the participants described that clinical pharmacists could provide a broad knowledge of medicine.

*“ She [the pharmacist] could answer it all… she is competent and knows what she is doing [regarding medication]”* [P10 Male].

#### Theme 2: mixed perceptions of a medication review

The majority of patients were pleased with the medication review.

*“It [medication review] is then to check whether what I get fits”* [P1 Female].

*“Maybe for research [use of information from medication review]… Can you [at the hospital] do something better than [the patient already receives of medication]…”* [P7 Female].

Some patients did not remember the exact content of the conversation with the pharmacists or the medication review, which was ascribed to forgetfulness due to tiredness.

*“…I don’t remember that anymore [what was talked about]…”* [P6 Female].

All patients were pleased pharmacists could share relevant information and medication recommendations with the respective GPs.

*“That’s perfectly fine!… That’s what you have to do today in the new world [sharing knowledge across sectors]”* [P11 Male].

#### Theme 3: satisfaction with the general outcome of the medication review

Most patients described that the overall pharmacist-led review provided a feeling of safety, mainly due to the communication between the patient and the pharmacist about the medication. Additionally, some of the patients suggested that the medication review had confirmed that the patients were being prescribed and treated with “the correct” medication.

*“It was just confirmation that I got the right [medication], but probably not all people get the right ones”* [P10 Male].

Many of the patients appreciated being listened to by the pharmacist and appreciated that they had time to review the medication thoroughly.

*“ Definitely [feels listened to during conversation]… There was no stress, she had time to talk to us [patient and relative], it was really nice. We both said that to each other when she had left, it was a really nice conversation”* [P9 Female + Relative Male].

There were mixed perceptions as to whether the medication review provided new knowledge.

*“Yes, I became wiser [after the conversation with the pharmacist]”* [P11 Male].

*“Nooo, I honestly don’t think so [gained new knowledge from talking to the pharmacist]… yes and no…”* [P1 Female].

#### Theme 4: safety perception with medication treatment

During hospitalization, the patients felt safe about their medication treatment, and most believed they had received sufficient information. However, some patients were not interested in learning about the new medication.

*“…I’m more comfortable with this [medication during hospitalization] than what I get from GP”* [P2 Female].

*“… really, I don’t need to know what kind of tablets [P1 receives during hospitalization]…”* [P1 Female].

Many patients were comfortable asking critical questions to the HCPs about the rationale of their medication treatment, and these critical questions also provided a feeling of safety during admission.

*“Yes, you sometimes do that [question whether the medication the patient receives is necessary]”* [P3 Female].

*“I’m the type, I ask first, what is it that you put in me [at the hospital], and what is it and what good does it do”* [P2 Female].

### Survey of HCPs

The questionnaire was sent to 54 HCPs from the orthopedic ward: 26 hospital-based physicians, 14 nurses, and 14 healthcare assistants. A total of 26 HCPs completed the questionnaire (response rate 48%), with responses from 10 hospital-based physicians (38%), eight nurses (31%), and eight healthcare assistants (31%).

Almost 85% of the HCPs (n = 22) were familiar with the pharmacist conducting medication reviews at the ward, including all 10 hospital-based physicians, whereas a few of the nurses (n = 2) and healthcare assistants (n = 2) were unfamiliar with the pharmacists’ role. Only 52% of the HCP’s had collaborated with the clinical pharmacist regarding a medication review (n = 12); among these were six hospital-based physicians. The HCPs who had collaborated with the clinical pharmacist reported that they were “very satisfied” or “satisfied” with the reviews (n = 10, 83%), whereas the remaining two participants were neither “dissatisfied” nor “satisfied”.

#### The impact of pharmacists’ and medication reviews on the orthopedic ward

**Fig. 2:**
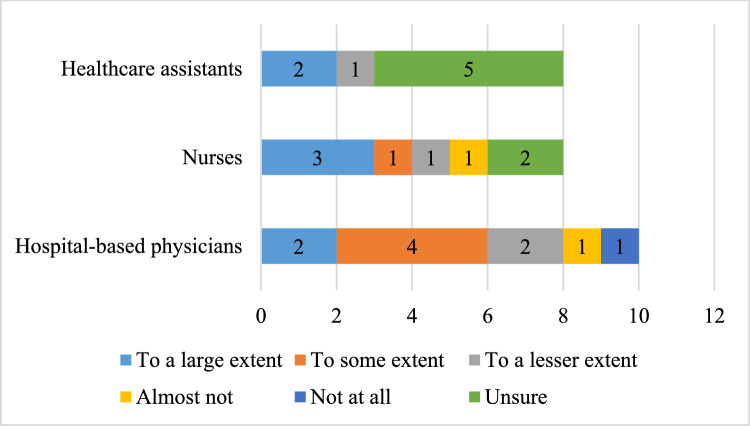
Distribution of ratings among HCPs in terms of medication reviews reliving their daily workload

Most HCPs believed that pharmacists on the ward generally have a positive impact. More than 70% of the HCPs reported that pharmacist-led medication reviews improved the quality of medication prescription, with the most positive ratings from hospital-based physicians (Table 1). Further, the service was reported to impact patient safety positively (Table 1). Nearly all HCPs found the presence of the pharmacist on the ward to increase their sense of security regarding the medication (Table 1). More than half of the hospital-based physicians and nurses reported that their work was relieved by the medication review, whereas most healthcare assistants were unsure of the impact (Fig. 2). However, corrections or questions about the medication can cause extra work for the HCPs.

**Table 1:** Display of ratings of the impact of pharmacists’ and medication reviews

	To a large extent	To some extent	To a lesser extent	Almost not/ not at all	Unsure
Do you believe that pharmacist-led medication reviews contribute to an increased quality of admitted patients’ use of medicine?	17 (65%)	2 (8%)	1 (4%)	0	6 (23%)
Do you believe that pharmacist-performed medication reviews have a positive impact on patient safety?	19 (73%)	5 (19%)	0	0	2 (8%)
To what extent have pharmacist-led medication reviews relieved your daily workload?	7 (27%)	5 (19%)	4 (15%)	3 (12%)	7 (27%)
Did the introduction of pharmacists on the ward have a positive impact?	22 (85%)	2 (8%)	0	0	2 (8%)
Does the presence of the pharmacists on the ward increase your sense of security regarding the medication treatment?	12 (46%)	11 (42%)	0	1 (4%)	2 (8%)

#### The visibility of the pharmacists’ recommendations from the medication review

Nearly all HCPs read the pharmacists’ written medication notes in the electronic chart (Table 2). In terms of the recommendations, nearly all (90%) hospital-based physicians report using the written recommendations. Conversely, only 40% of hospital-based physicians use pharmacists’ recommendations after oral face-to-face delivery (Table 2).

**Table 2:** Display of ratings of the visibility of the pharmacist recommendations from the medication review

	To a large extent	To some extent	To a lesser extent	Almost not/ not at all	Unsure
Do you read the medication notes in the electronic chart from the pharmacists?	7 (27%)	8 (31%)	8 (31%)	3 (12%)	0
Do *hospital-based physician*s apply the suggestions from pharmacists’ medication reviews from the notes in the electronic chart?	6 (60%)	3 (30%)	0	0	1 (10%)
Do *hospital-based physicians* apply the suggestions from the pharmacists’ medication review at/after an oral discussion?	3 (30%)	1 (10%)	1 (10%)	2 (20%)	3 (30%)

## Discussion

### Statement of key findings

Although patients perceived the meeting with the pharmacist positively, they expressed mixed perceptions of the impact of the pharmacist-led medication review. Both patients and HCPs were satisfied with the general outcome of the medication reviews in terms of quality and safety, and the reviews relieved nearly half of the HCPs’ daily workload.

### Strengths and weaknesses

One strength of this pilot study is that the mixed method design provided various insights that evaluated the implementation of pharmacist-led medication reviews for older admitted patients at an orthopedic ward. Individual interviews allow patients to describe and explain their experiences and attitudes towards pharmacist-led medication reviews. This detailed description constitutes an important element in fulfilling various trustworthiness criteria, such as the transferability criterion proposed by Guba and Lincoln [32, 33]. Further, two researchers coded the interviews and discussed discrepancies before reaching analytical agreement. Thus, the investigative triangulation approach ensured high data quality. In addition, the study met the authenticity quality criterion of fairness because the same interview guide was applied during all interviews, and participants were treated equally [32, 33]. Using two validated questionnaires from previous research studies ensured that data collection was reliable [34]. The questionnaires enabled data collection from a broad group of HCPs, ensuring participant diversity [19].

However, there are also some limitations to the pilot study. First, the findings may not fully represent all admitted patients, as it was only possible to interview a limited number of patients. However, this study intended to explore different perspectives from those affected by the pharmacist-led medication reviews rather than report all patients’ perspectives. In addition, we could only include patients for the interview if they were assessed as capable of contributing sufficiently. This inclusion criteria may have introduced a bias as only the best-functioning patients could be interviewed. The credibility of the thematic analysis is challenged as patients did not validate the outcomes, which is another study limitation [33]. Despite multiple reminders, not all hospital-based physicians, nurses and healthcare assistants responded to the questionnaire, which resulted in a response rate of 48%. This is relatively low compared to other studies that report response rates from 60 to 81% [6, 9, 12]. However, the low response rate in our study could be attributed to the short data collection period (May 2024) or lack of time or interest from the HCPs. The low response rate may be due to HCPs who have negative opinions of medication reviews and were unwilling to participate, introducing bias. Lastly, no information was collected regarding the demographics of the HCPs, such as age, sex, or years of service. These factors could have influenced the HCPs’ perspectives on medication reviews, for example, a junior physician may value consultation with a pharmacist more than a senior physician.

### Interpretation

All patients and HCPs were generally positive towards the communication skills of pharmacists at the orthopedic ward. Particularly, all patients were comfortable during the patient interviews with the pharmacists, where they felt listened to and acknowledged, and the pharmacists communicated understandably. Furthermore, the patients particularly appreciated the extra time and quality assessment provided about their medication. Similar findings have been reported earlier [6, 24], where one study explored pharmacists’ and patients’ views on effective pharmacist-patient communication [24]. Here, the patients reportedly preferred simple directions and easy-to-understand language without complex medical terms, and they valued prioritized time for medication reviews. Patients could ask questions and discuss their medication [24].

Nearly all HCPs were familiar with pharmacists on the orthopedic ward, and the HCPs in this study reported that they were “very satisfied” or “satisfied” with the medication reviews. A study investigating the impact of pharmacist-led medication reconcilliation and review for patients in an orthogeriatric setting showed a significantly lower rate of drug-related problems after discharge [25]. In addition, the pharmacists provided important medication support to elderly patients after discharge, emphasizing the importance of the pharmacist’s role in managing patient care and the safe use of medication [25]. Questionnaire responses from HCPs and patient interviews support the positive impact of pharmacist-led medication reviews on patient safety in the medication process.

Some patients reported difficulties recalling the purpose of the medication review and the exact conversation with the pharmacist. The patients who had received a medication review previously had a greater understanding of the purpose. However, medication reviews were valued differently. Some reflected that the review was a simple check-up of the medication lists, whereas others described how it provided them with new knowledge about their medication. These findings are consistent with a Swedish study, where patients had difficulty retaining information from pharmacists during a medication review [17]. In another similar study, recall problems were reported even after the patients had received structured medication counselling upon discharge [26]. An explanation for the recall problem is that patients have contact with different HCPs during hospitalization, adding confusion to the information provided. Further, as all patients had undergone surgery, a general anesthetic and were prescribed opioids after surgery, their ability to recall the medication review may have been affected. General anesthesia is reported to cause problems with retaining and recalling new information during the recovery period [27, 28]. Hence, information should be given to patients as close as possible to discharge time, preferably in writing [27]. Our findings support these studies, resulting in a rethinking of the optimal timing for providing medication information.

After implementing pharmacist-led medication reviews, the HCPs reported high satisfaction with the quality and patient safety of the medication regimens of admitted patients. Past studies report satisfaction from HCPs and a high degree of patient contentment concerning pharmacist-led interventions [6, 9, 12]. For example, hospital physicians rated pharmacist-led medication reviews as the most valued and supported activity [6]. Similarly, 94% of medical and nursing staff in an orthopaedic surgery unit would not return to the previous organization without ensuring clinical pharmacists’ presence on the ward [9]. Further, the study reported that the pharmacists’ skills were an advantage and improved patient care, quality and drug-related patient safety [9, 12].

Nearly all patients felt safe about their medication treatment during admission after the interview with the pharmacists. Similarly, one study reported that patients feel safe and well-informed about their medicines after pharmacist-led medication counselling sessions with patients before discharge [6]. Thus, pharmacists are important in patient care around patients’ medication.

Further, contributions from the pharmacists included easing the workload of nurses and medical staff, thus allowing them to dedicate more time to their key roles [6, 9]. A Swedish study reported that implementing a comprehensive pharmacist-led medicine management model decreased the time by at least 1 h spent by HCPs with each admitted patient [29]. However, in our study, only half of the HCPs (half were hospital-based physicians) reported that pharmacist-led medication reviews relieved their daily workload. In contrast, 25% of HCPs disagreed with this statement. Possible explanations from this divergence compared to the literature could be that the primary purpose of introducing pharmacist-led medication reviews is to support hospital-based physicians in the medication process. Thus, the workload of nurses and healthcare assistants may be less eased compared to that of hospital-based physicians. However, nearly half of the hospital-based physicians did not feel that the pharmacists relieved their workload, which may be due to extra time spent reading potential pharmacist recommendations in the electronic chart, subsequently requiring decision-making about medicines. Conversely, hospital-based physicians preferred using the written notes in EPJ compared to a face-to-face delivery of recommendations, contrasting other results related to the acceptance of pharmacist recommendations [12, 30, 31]. A high acceptance rate of medication reviews can be related to the delivery of pharmacist recommendations face-to-face instead of as written recommendations [12, 31].

### Future research

Future research should focus on specific considerations for patients admitted to the orthopeadic ward. At this ward, the orthopeadic physicians may not be as accessible to physicians from the medical department due to surgery, which limits the availability of the clinical pharmacist regarding the results of the medication review conducted. Hence, further studies should focus on the timing of the medication review to include optimal communication between clinical pharmacists and physicians.

Additionally, it is important to identify which patients would benefit most from a medication review with limited resources. Understanding which individuals would benefit most from a medication review ensures that the available resources are used efficiently and that patients most in need of this intervention are prioritized.

## Conclusion

The service is considered well-implemented because 85% of the HCPs reported being familiar to the introduction pharmacist-led medication reviews in the ward. Further, high satisfaction with the outcome of the medication reviews in terms of quality, patient safety, and positive impact on the ward from the perspectives of patients and HCPs suggests that the service is of high value. Although, pharmacists did not consistently ease the workload of HCPs, optimal delivery of pharmacist recommendations diverged from that suggested in the literature (written versus oral).

## Supplementary Information

Below is the link to the electronic supplementary material.

Supplementary file1 (PDF 246 KB)

Supplementary file2 (PDF 395 KB)
