# Supplementary material for: Patient and hospital staff perspectives on introducing pharmacist-led medication reviews at an orthopedic ward: a mixed methods pilot study
Source: Int J Clin Pharm. 2025 Feb 20;47(3):794–802. doi: 10.1007/s11096-025-01874-7 (PMC12125125; doi:10.1007/s11096-025-01874-7)
Supplement: Supplementary file 1 — Supplementary file1 (PDF 246 KB) [file 11096_2025_1874_MOESM1_ESM.pdf]

Supplementary Information S2

Introducing pharmacist-led medication reviews at an orthopaedic ward – perceived value from the perspectives of patients and hospital-based physicians, nurses and healthcare assistants - International Journal of Clinical Pharmacy,

Joo Hanne Poulsen Revell[1,2,3], Maja Schlünsen[1,2]*, Abisha Kandasamy[4], Annette Meijers[1,2], Jens Eggers[5], Lene Juel Kjeldsen[1,2,3]

Author's affiliations:

[1]The Hospital Pharmacy, University Hospital of Southern Denmark, Denmark
[2]The Hospital Pharmacy Research Unit, University Hospital of Southern Denmark, Denmark
[3]The Department of Regional Health Research, University of Southern Denmark, Odense, Denmark
[4]The Faculty of Health Sciences, University of Southern Denmark, Odense, Denmark
[5]Department of Orthopaedics, University Hospital of Southern Denmark, Denmark

***Corresponding author**

Maja Schlünsen**,** Email: Maja.Schlunsen@rsyd.dk

Interview guide to admitted patients at the orthopedic ward

Introduction to the purpose of the interview and framework of the interview

1. The purpose of the interview: explore patients' experience of a pharmacist-led medication review with focus on value and safety of the medication treatment.
   a. Brief presentation of the interviewer

2. The framework of the interview
   a. Time frame is 15-20 minutes including the introduction.
   b. The interviewer does not answer any drug-related questions, but possible questions can be passed on to the pharmacists or should be addressed to the hospital-based physician
   c. Make the participant aware that…
      i. recording of the interview using a dictaphone
      ii. the participants' rights in research and their anonymity
      iii. the possibility of withdrawing their consent
      iv. signing the consent form

Subject 1: The patient's experience of a medication review

You have had a medication review performed by a clinical pharmacist during your admission.

1) How was it talking to the pharmacist?
   a. Did the pharmacist listen?
   b. Was the conversation understandable?

2) How would you describe the purpose of a medication review?

3) Is this the first time, you have had a medication review performed by a pharmacist? (Yes No)

4) What do you think about your medication being reviewed by a pharmacist?

5) Is there anything else you think I should know or anything else you would like to share about your medication review experience?

Subject 2: The patients' sense of security in the medical treatment

6) Do you feel comfortable/safe with your medication treatment during hospitalization? (Yes No)
    a. If the answer is no:
        i. Did you feel comfortable with your medication treatment before admission?
        ii. Did the medication review make you feel more safe with your medicine?
        iii. Can you put some words to what is needed for you to feel safe?

    b. If the answer is yes:
        i. How has the medication review contributed to the safety around medicine?

Subject 3: Patient-perceived value of a medication review

7) In your words, what was your personal gain from talking to the pharmacist about your medicine?

8) How has the medication review contributed to your understanding of your medication during hospitalization?

9) You have been prescribed new medicine during hospitalization. Have you been informed about the new medicine? (Yes No)

    a. What counselling have you received about the new medicine?
    b. Did you receive sufficient information to take manage your medication after discharge?
    c. Is there some medication information you feel that you are missing?

10) What do you think, the information from the medication review will be used for?

11) How do you feel about important information from the pharmacists is shared with your GP?
