# Supplementary material for: Patient and hospital staff perspectives on introducing pharmacist-led medication reviews at an orthopedic ward: a mixed methods pilot study
Source: Int J Clin Pharm. 2025 Feb 20;47(3):794–802. doi: 10.1007/s11096-025-01874-7 (PMC12125125; doi:10.1007/s11096-025-01874-7)
Supplement: Supplementary file 2 — Supplementary file2 (PDF 395 KB) [file 11096_2025_1874_MOESM2_ESM.pdf]

Supplementary Information S3

Introducing pharmacist-led medication reviews at an orthopaedic ward – perceived value from the perspectives of patients and hospital-based physicians, nurses and healthcare assistants - International Journal of Clinical Pharmacy,

Joo Hanne Poulsen Revell[1,2,3], Maja Schlünsen[1,2]*, Abisha Kandasamy[4], Annette Meijers[1,2], Jens Eggers[5], Lene Juel Kjeldsen[1,2,3]

Author's affiliations:

[1]The Hospital Pharmacy, University Hospital of Southern Denmark, Denmark
[2]The Hospital Pharmacy Research Unit, University Hospital of Southern Denmark, Denmark
[3]The Department of Regional Health Research, University of Southern Denmark, Odense, Denmark
[4]The Faculty of Health Sciences, University of Southern Denmark, Odense, Denmark
[5]Department of Orthopaedics, University Hospital of Southern Denmark, Denmark

***Corresponding author**

Maja Schlünsen**,** Email: Maja.Schlunsen@rsyd.dk

Survey regarding pharmacist-led medication reviews at the orthopedic wards at the University Hospital of Southern Denmark.

Purpose: to explore your perception on pharmacist-led medication reviews at the ward with specific focus on quality and safety of your medication treatment. (Please indicate your answer in the tick box)

1. I am a…

☐ Hospital-based physician ☐ Nurse ☐ Healthcare assistant

1.a. For hospital-based physicians only. What is your current position?

☐ Chief physician ☐ Department physician ☐ KBU physician ☐ Other

2. Do you know that pharmacists have conducted medication reviews at the ward (talking to the patient about their medications)? (Tick the box)

☐ Yes ☐ No ☐ Not sure.

2.a. If yes, did you collaborate with the pharmacists regarding a medication review? (Tick the box)

☐ Yes ☐ No ☐ Not sure.

2.b. If yes, how satisfied are you with the execution/conduct of pharmacist-performed medication reviews at the ward? (Tick the box)

Very satisfied	Satisfied	Neither	Unsatisfied	Very dissatisfied	Not sure

3. Do you believe that pharmacist-led medication reviews contribute to an increased quality of admitted patients' use of medicine?

To a large extent	To some extent	To a lesser extent	Not at all	Not sure
Please elaborate:				

4. Do you believe that pharmacist-performed medication reviews have a positive impact on patient safety? (Tick the box)

To a large extent	To some extent	To a lesser extent	Not at all	Not sure

5. To what extent have pharmacist-led medication reviews relieved your daily workload? (Tick the box)

To a large extent	To some extent	To a lesser extent	Not at all	Not sure

6. Did the introduction of pharmacists on the ward have a positive impact? (Tick the box)

To a large extent	To some extent	To a lesser extent	Not at all	Not sure

7. Does the presence of the pharmacist on the ward increase your sense of security regarding the medication treatment? (Tick the box)

To a large extent	To some extent	To a lesser extent	Not at all	Not sure

8. Do you read the medication notes in the electronic chart from the pharmacists? (Tick the box)

To a large extent	To some extent	To a lesser extent	Not at all	Not sure

8.a. For hospital-based physicians only. Do you apply the suggestions from pharmacists' medication reviews from the notes in the electronic chart? (Tick the box)

To a large extent	To some extent	To a lesser extent	Not at all	Not sure

8.b. For hospital-based physicians only. Do you apply the suggestions from the pharmacists' medication review at/after an oral discussion? (Tick the box)

To a large extent	To some extent	To a lesser extent	Not at all	Not sure

9. What potential changes do you think will improve pharmacist-performed drug reviews at the ward?

10. For hospital-based physicians only. What other patient groups (besides patients with hip fractures), do you think, would benefit from a pharmacist-performed medication review at the ward?

Thank you very much for your reply.
